# Cytological grading of breast cancers and comparative evaluation of two grading systems

**DOI:** 10.4103/0970-9371.70738

**Published:** 2010-04

**Authors:** Farooq Ahmed Wani, Subhash Bhardwaj, Dinesh Kumar, Pervez Katoch

**Affiliations:** Senior Resident, SKIMS Medical College, Srinagar, India; 1 Department of Pathology, Government Medical College, Jammu, India; 2 Department of Preventive and Social Medicine, Government Medical College, Jammu, India; 3 Department of Pathology, Government Medical College, Jammu, Jammu and Kashmir, India

**Keywords:** Breast cancer, cytological grading, chemotherapy

## Abstract

**Aim:**

To evaluate and compare the cytograding of breast cancers using Robinson’s and Mouriquand’s grading methods.

**Materials and Methods:**

A 5-year retrospective (from Oct 2000 to Sept 2005) and 1-year prospective study (from Oct 2005 to Oct 2006). A total of 110 fine-needle aspiration cytology (FNAC) cases of breast cancers were studied. These were graded according to Robinson’s and Mouriquand’s grading methods (grade I–III) followed by comparison of the two methods.

**Results:**

Of the 110 cases graded according to Robinson’s method, 28 (25.45%) cases were grade I, 46 (41.81%) grade II, and 36 (32.72%) were grade III, whereas using Mouriquand’s grading methods, 28 (25.45%), 42 (38.18%), and 40 (36.36%) cases were graded as grade I, II, and III, respectively. A high degree of concordance was observed between the two grading methods (90.9%). A highly significant relationship between the scores obtained by two methods was also observed (*P*=0.004).

**Conclusions:**

A comprehensive cytological grading of breast cancers is possible by using two different methods proposed by Robinson and Mouriquand. In spite of a high degree of concordance between the two methods, the Robinson’s grading system has been found to be easier and better because of more objective set of criteria and easy reproducibility.

## Introduction

Breast cancer is the second most common type of cancer and the second leading cause of cancer-related deaths in females all over the world.[1] Fine-needle aspiration cytology (FNAC) is being increasingly used to determine the benign and malignant nature of the lesions and many studies have shown that this technique can provide additional information about the intrinsic features of the tumors as well as their prognosis.[2] Grading of breast carcinoma, while the tumor is still in vivo, would be the most ideal and desirable situation, as it would be helpful in the selection of patients for appropriate therapy.[3] As neoadjuvant therapy, including preoperative chemotherapy and tamoxifen, is becoming increasingly common for early breast cancer, it is desirable to grade tumors before surgery so that the most appropriate medical regime can be selected.[4]

The present study used Robinson’s and Mouriquand’s methods for the grading of breast carcinomas followed by comparison of the two grading methods. Correlation between cytological grading and clinical status at the time of FNAC was also evaluated.

## Materials and Methods

Ours was a 5-year retrospective and 1-year prospective study. After obtaining clearance from the institutional ethics committee, all records regarding diagnosed or highly suspicious breast cancer cases (for the period starting from October 2000) were retrieved from the cytopathology section. These records were then matched with the database of breast cancers registered by the department of radiotherapy and surgical operation log books. Patients diagnosed at other institutions but receiving treatment in the hospital were excluded from the study. Retrospective study material comprised 90 cases, which were diagnosed as carcinoma breast or highly suspicious for malignancy on FNAC from October 2000 to September 2005. Prospective study material comprised 20 new cases diagnosed as breast cancer or suspicious of breast cancer in the course of 1 year from October 2005 to October 2006. These cases were either referred from the department of surgery or radiotherapy for confirmation of diagnosis. All the patients referred were subjected to FNAC. The smears were stained with May–Grünwald–Giemsa and Papanicolaou stains.

Grading of breast carcinoma was done according to Robinson’s and Mouriquand’s methods by two independent observers. Robinson’s method takes into account the following criteria: cell dissociation (clusters/single cells), cell size (1–2/3–4/≥5×RBC size), cell uniformity (monomorphic/mildly pleomorphic/pleomorphic), nucleoli (indistinct/noticeable/prominent), nuclear margins (smooth/folds/buds or tufts) and nuclear chromatin (vesicular/granular/clumped or cleaved). Each of the above criteria was given scores 1–3 and total sum of scores of all the criteria were used to grade the tumors.[4,5] Based on the above-mentioned criteria, breast cancers were graded into grade I (score 6–11), grade II (score 12–14) and grade III (score 15–18), respectively.

Mouriquand’s method takes into account the following criteria: cellular characters (clustering, 0/isolated cells, 3), nuclear features (anisokaryosis, 2/large size, 3/budding, 2/naked, 3/hyperchromasia, 2/hypochromasia, 3), nucleoli (blue, 2/red, 3) and number of mitoses (≥3/slide=1, ≥6/slide=3).[6] Based on these criteria, breast cancers were graded into grade I (score<5), grade II (score 5–9) and grade III (score ≥10).

### Statistical analysis

The data were analyzed with the help of computer software Microsoft Excel for Windows and Epi-Info Version 6.0, CDC, Atlanta, GA. Grading reported qualitatively in terms of percentages. The agreement between the methods was assessed by the use of Kappa statistics. Correlation between the scores evaluated using Spearman rank correlation and its significance evaluated using “*t*” test. A *P* value of less than 0.05 was considered as statistically significant. All *P* values used were two tailed.

## Results

**Figure 1:**
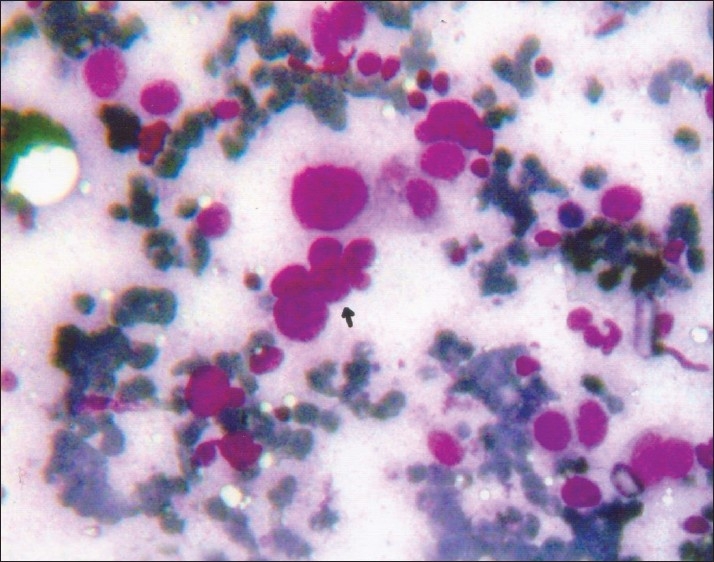
Photomicrograph of grade III breast cancer showing nuclear budding as indicated by arrow (MGG, ×400)

Of the 110 cases graded according to Robinson’s method, 28 (25.45%) cases were grade I, 46 (41.81%) grade II and 36 (32.72%) cases were grade III tumors, whereas using Mouriquand’s method, 28 (25.45%) cases were grade I with scores <5, 42 (38.18%) were grade II with scores 5–9 and 40 (36.36%) cases were grade III with scores>10 [Table 1, Figure 1].

**Table 1:** Comparison of Robinson’s and Mouriquand’s grading

Mouriquand’s grading	Robinson’s grading			Total
	I	II	III	
I	26	2	–	28 (25.45)
II	2	39	1	42 (38.18)
III	–	5	35	40 (36.36)

Total	28 (25.45)	46 (41.81)	36 (32.72)	110 (100)

Unweighted kappa=0.8615, SE=0.0424, 95% confidence limits=0.7783–0.9447: figures in parentheses are in percentage

Of the 28 cases graded as grade I by Robinson’s method, 26 were graded as grade I, whereas two cases were over graded as grade II by Mouriquand’s method. Most of the discordance was seen in grade II tumors. Of the 46 (41.81%) cases graded as grade II by Robinson’s method, 39 cases were graded as grade II, whereas two cases were under-graded as grade I and five cases were over-graded as grade III by Mouriquand’s method. Of the 36 cases graded as grade III by Robinson’s method, 35 cases were graded as grade III by Mouriquand’s method and only one case was under-graded as grade II [Table 1].

A high degree of concordance between Robinson’s and Mouriquand’s method was observed in our study. There was total agreement in 90.9% of the cases. Correlation of the scores obtained by Robinson’s and Mouriquand’s method is shown in Table 2. Our study showed a highly significant relationship between the scores obtained by Robinson’s and Mouriquand’s methods (*P*=0.004).

**Table 2:** Correlation * observed in scores obtained by Robinson’s method and Mouriquand’s method

Criteria	Robinson’s scoring	Mouriquand’s scoring
Minimum score	8	2
Maximum score	17	16
Mean score	13.12	8.027
Standard deviation	2.46	3.89
Standard error	0.235	0.371

* Spearman’s Rho. r=0.95 t(108) *P*=0.004 (two-tailed), highly significant

## Discussion

Breast cancer is the second most prevalent cancer among Indian women, the first being cervical cancer.[7] In India, the breast is reported as the most common site of cancer in Mumbai and Thiruvanathapuram, whereas it is the second most common site of cancer in Chennai and Dibrugarh. In Bangalore, it is ranked third according to data reported from hospital-based cancer registries (ICMR).[8]

The purpose of cytoprognostic grading in breast cancers is to identify fast growing tumors (grade III), which are more likely to respond to chemotherapy than low grade, slow growing tumors, which may be better suited to pre-treatment with tamoxifen. Assessment of biological aggressiveness by cytological grading without removing the tumor would, therefore, be of immense value.[9]

In the present study, we found predominance of grade II tumors, which is in corroboration with those of previous studies. Robinson *et al*.[4] reported grade II tumors (44%) comprising the largest group.

Chhabra *et al*.[10] reported maximum number of cases as grade II (52%) followed by grade I (30%) and grade III (18%). Robles *et al*.[2] also reported predominance of grade II tumors (39%) followed by grade I (33%) and grade III (28%). However, in our study, grade III tumors formed the second largest group. The most probable reason for the grade III tumors forming the second largest group could be the late presentation of the patients in our setup.

In our study, Mouriquand’s grading showed a predominant grouping of cases into grade II (38.18%), consistent with the findings reported by Pandit and Parekh.[11]

Not many studies directly comparing the two grading methods could be found in the literature. Only one study conducted by Das *et al*.[12] was retrieved from the literature, where they found total agreement in grading by both the methods in 76.9% cases. In our study, we found a high degree of concordance between Robinson’s and Mouriquand’s cytograding methods. There was a total agreement in 90.9% of the cases.

However, few studies comparing different cytograding methods have shown similar results. In the study done by Bhargava *et al*.,[13] comparing Robinson’s, Fischer’s modification of Black’s nuclear grading and Scarff-Bloom-Richardson methods, Robinson’s grading system was found to have best correlation with histopathology grades as well as Estrogen Receptor/Progesterone Receptor expression.

Some degree of discordance between the two grading methods was observed in all the grades with the majority of the discordant cases observed in grade II tumors. Of the 28 (25.45%) cases graded as grade I by Robinson’s method, 26 cases were graded as grade I, whereas two cases were over-graded as grade II by Mouriquand’s method. Of the 46 (41.81%) cases graded as grade II by Robinson’s method, 39 cases were graded as grade II by Mouriquand’s method, whereas five cases were over-graded as grade III and two cases were under-graded as grade I. The reason for the over-grading of the tumors by the Mouriquand’s method appears to be the presence of mitosis in most of the discordant cases. Mitosis is one of the parameters used for grading by Mouriquand’s method, whereas mitosis is not taken into consideration in Robinson’s method. In the study done by Das *et al*.,[12] all the discordant cases were in grade II tumors but a minor degree of discordance was also observed in grade I and grade III tumors. Our study showed a highly significant relationship between the scores obtained by Robinson’s and Mouriquand’s methods (*P*=0.004).

## Conclusions

A comprehensive cytological grading of the breast cancers is possible by using two different methods proposed by Robinson and Mouriquand. In spite of a high degree of concordance between the two grading methods, the grading of breast cancers by Robinson’s method has been found to be easier and better because of the more objective set of criteria and easy reproducibility.

It is, however, important to draw the readers’ attention to the fact that the conclusions drawn might have been affected by selection and measurement bias to some extent, in spite of adequate efforts having been made at the design stage to minimize the distortion in the estimates. Thus, it is suggested that a conscious effort should be made to include the cytological grading in all the FNAC reports of breast cancers, so that an appropriate decision regarding the preoperative neoadjuvant therapy can be taken and the over-treatment of low-grade cancers is avoided.
